# Monocyte Chemotactic Protein 1-Induced Protein 1 Is Highly Expressed in Inflammatory Bowel Disease and Negatively Regulates Neutrophil Activities

**DOI:** 10.1155/2020/8812020

**Published:** 2020-12-22

**Authors:** Jian Lin, Gengfeng Li, Chunjin Xu, Huiying Lu, Cui Zhang, Zhi Pang, Zhanju Liu

**Affiliations:** ^1^Department of Gastroenterology, The Shanghai Tenth People's Hospital of Tongji University, Shanghai, China; ^2^Department of Gastroenterology, Affiliated Hospital of Putian University, Putian, China; ^3^Department of Gastroenterology, The First People's Hospital of Shangqiu City Affiliated to Xinxiang Medical University, Shangqiu, China; ^4^Department of Gastroenterology, Suzhou Municipal Hospital Affiliated to Nanjing Medical University, Suzhou, China; ^5^Department of Gastroenterology, The Second Affiliated Hospital of Zhengzhou University, Zhengzhou, China

## Abstract

Monocyte chemotactic protein 1-induced protein 1 (MCPIP-1) is highly expressed in activated immune cells and plays an important role in negatively regulating immune responses. However, its role in regulating neutrophil functions in the pathogenesis of inflammatory bowel disease (IBD) is still unclear. Here, we found that MCPIP-1 was markedly increased at both the transcriptional and translational levels in inflamed mucosa of IBD patients compared with healthy controls, which was mainly expressed in neutrophils. Interestingly, MG-132, a proteasome inhibitor reducing the degradation of MCPIP-1, further facilitated neutrophils to express MCPIP-1 *in vitro*. Importantly, MCPIP-1 markedly downregulated the production of ROS, MPO, and proinflammatory cytokines (e.g., interleukin-1*β*, interleukin-6, tumor necrosis factor-*α*, interleukin-8, and interferon-*γ*) and suppressed the migration of IBD neutrophils. Consistently, the same functional changes were observed in neutrophils from mice with myeloid-targeted overexpression of MCPIP-1 as MG-132 did. Altogether, these findings suggest that MCPIP-1 plays a negative role in regulating neutrophil activities through suppressing the production of ROS, MPO, and proinflammatory cytokines and inhibiting the migration. MG-132 may partially modulate the function of neutrophils via the induction of MCPIP-1. Therefore, targeting MCPIP-1 or exogenous supplementation of MG-132 may provide a therapeutic approach in the treatment of IBD.

## 1. Introduction

Inflammatory bowel disease (IBD), including Crohn's disease (CD) and ulcerative colitis (UC), is a chronic inflammatory disease that affects the gastrointestinal tract. With the characteristics of remittent and progressive inflammatory disorders, IBD has long been regarded as a risk factor of colon cancer [1]. The incidence of IBD in China keeps ascending in past years, which causes a heavy economic burden for the country [2]. Although the etiology and pathology of IBD are still not fully understood, it is generally considered that anomalous immune response to intestinal microbiota involves the pathogenesis [3–6]. As a feature of dysregulated intestinal immune responses in IBD, increasing numbers of neutrophils are observed to accumulate in the affected mucosa and serve as an indispensable effector in the pathogenesis [7].

Neutrophils are short-lived effectors but are the most abundant immune cells in the peripheral blood, which function as crucial effector cells of the innate immune system and a double-edged sword in intestinal immunity [8]. As the first line of host defense against the invasion of invading microbes, neutrophils migrate to inflammatory sites under inflammatory conditions [9]. They eliminate invading microorganisms through phagocytosis, the release of antibacterial peptides (e.g., cathepsin, defensin, and calprotectin) from granules, the production of reactive oxygen species (ROS) and myeloperoxidase (MPO), and the formation of neutrophil extracellular traps (NETs) [10–12]. Neutrophils also play an important role in intestinal mucosal healing through producing vascular endothelial growth factor (VEGF), tissue growth factor- (TGF-) *β*, and matrix metalloproteinase (MMP), inducing the accumulation of double-strand break and releasing extracellular vesicles or microparticles [13, 14]. However, extravasation of neutrophils from peripheral blood to inflamed intestinal mucosa is related to the disease activity of IBD patients [15], and excessive production of ROS may cause tissue damage [16]. Moreover, neutrophils are also the main source of proinflammatory cytokines (e.g., interleukin- (IL-) 1*β*, IL-6, tumor necrosis factor- (TNF-) *α*, and interferon- (IFN-) *γ*) and chemokines (e.g., IL-8) that recruit more neutrophils and monocytes into the infected sites [17]. Recently, we have reported that CD177^+^ neutrophils as functionally activated neutrophils negatively regulate IBD through producing IL-22, an important protective cytokine that guarantees epithelial barrier integrity [18]. Our other study further demonstrated that proinflammatory activities of mucosal neutrophils are inhibited in IBD patients after anti-TNF-*α* mAb therapy [19]. Although neutrophils regulate intestinal homeostasis and are involved in the pathogenesis of IBD in several ways, the underlying mechanisms whereby neutrophils regulate intestinal mucosal immune responses in IBD are still not clear.

Monocyte chemotactic protein 1-induced protein 1 (MCPIP-1), also known as regnase-1, is a novel zinc finger protein encoded by the *ZC3H12A* gene [20], which is expressed in a variety of immune cells (e.g., monocyte, macrophage, and CD4^+^ T cells) and increased by several inflammatory stimuli such as monocyte chemotactic protein 1 (MCP-1), ligands of toll-like receptors (TLR), TNF-*α*, and IL-1*β* [21]. Originally, MCPIP-1 is found as a negative regulator in regulating immune response of macrophages. With the function of deubiquitination, it removes ubiquitin moieties attached to proteins such as TNF receptor associated factor (TRAF)2, TRAF3, and TRAF6 and subsequently suppresses c-Jun N-terminal kinase (JNK) and nuclear factor kappa-B (NF-*κ*B) signaling pathways [22]. The accumulation of MCPIP-1 could inhibit the activity of the NF-*κ*B pathway, leading to restricting the synthesis of MCPIP-1 itself [23], and it acts as an endonuclease that degrades the mRNA of proinflammatory cytokines, such as IL-6, IL-1*β*, IL-12, IL-2, TNF-*α*, and the mRNA of itself [21, 23, 24]. In addition, MCPIP-1 also degrades and inhibits the biosynthesis of numerous microRNAs (miRs) (e.g., miR-135b, miR-146a, miR-21, miR-155, miR-143, and miR-145) [25] and plays a negative regulator in the proliferation and differentiation of T cells and tumor cells [26, 27]. Under physiological conditions, MCPIP-1 keeps at a relatively low level in immune cells and involves the maintenance of immune homeostasis. However, under inflammatory conditions, such as septic shock or autoimmunity, MCPIP-1 is increased and then plays an important role in downregulating the inflammatory procedure as through suppressing NF-*κ*B signaling pathways and degrading the mRNA of proinflammatory cytokines. Consistently, evidence has shown that MCPIP-1-deficient mice suffer severe systemic inflammation characterized by T and B cell overactivation and are vulnerable to septic shock [28, 29]. Although MCPIP-1 functions as a “brake” to aberrant activation of the immune system, the role of MCPIP-1 in regulating the function of neutrophils remains unknown.

In the current study, we found that MCPIP-1 was markedly increased at both the transcriptional and translational levels in inflamed mucosa of patients with IBD compared with that in healthy controls and that it was mainly expressed in neutrophils. Furthermore, overexpression of MCPIP-1 in neutrophils induced *in vitro* by MG-132, a proteasome inhibitor that induces overexpression of MCPIP-1 in several cell types by reducing the degradation of MCPIP-1, markedly suppressed the production of ROS, MPO, and proinflammatory cytokines, and the migration. Consistently, the same functional alterations were observed in Mcpip^Mye-tg^ neutrophils as MG-132 did. These results thus indicate that MCPIP-1 as a critical regulator plays an important role in modulating the functions of neutrophils in IBD.

## 2. Materials and Methods

### 2.1. Patients

All patients with IBD were recruited from the Department of Gastroenterology, the Shanghai Tenth People's Hospital of Tongji University (Shanghai, China) from February 2018 to October 2019. EDTA-anticoagulated blood samples (15-20 mL) were obtained from patients with active CD (CD, *n* = 22), patients with active UC (UC, *n* = 24), and healthy controls (HC, *n* = 27) after overnight fasting. Colon biopsy samples were obtained from patients with active CD (*n* = 14) or UC (*n* = 12) and HC (*n* = 10) who underwent endoscopy. The clinical characteristics of these patients with IBD are shown in Table 1. The diagnoses for IBD were based on clinical characteristics, radiological and endoscopic examination, and histological findings. International standard criteria such as Crohn's disease activity index (CDAI) and Mayo scores were used to assess the severity of disease in patients with CD and UC, respectively [30]. This study was approved by the Institutional Review Board for Clinical Research of the Shanghai Tenth People's Hospital of Tongji University.

### 2.2. Mice

Specific pathogen-free C57BL/6J mice with myeloid-targeted overexpression of MCPIP-1 (namely Mcpip^Mye-tg^) were kindly provided by Drs. Jianli Niu and Pappachen Kolattukudy from the Burnett School of Biomedical Science, College of Medicine, University of Central Florida (Orlando, FL, USA). Mcpip^Mye-tg^ mice were generated using the protocol as described previously [31]. C57BL/6J wild-type (WT) mice were purchased from the Shanghai SLAC Laboratory Animal Co., Ltd. (Shanghai, China). These mice were raised under specific pathogen-free conditions in microisolator cages with filtered air and were fed autoclaved food and water at the animal facility of the Tongji University. All mice for experiments were 20−25 g of weight and aged 8–10 weeks. Animal studies were reviewed and approved by the Institutional Animal Care and Use Committee of the Tongji University.

### 2.3. Materials

Cell culture reagents including RPMI-1640 medium, fetal bovine serum (FBS), streptomycin and penicillin, 2-mercaptoethanol, and phosphate-buffered saline (PBS) were purchased from HyClone (Logan, UT, USA). Phorbol 12-myristate 13-acetate (PMA), ionomycin, and lipopolysaccharide (LPS) were purchased from Sigma-Aldrich (St. Louis, MO, USA). MG-132 was purchased from MCE (Monmouth Junction, NJ, USA). Amplex Red Hydrogen Peroxide Assay Kit for measuring the level of ROS or MPO was purchased from Thermo Fisher (Carlsbad, CA, USA). Enzyme-linked immunosorbent assay (ELISA) kits for cytokines were purchased from BioLegend (San Diego, CA, USA).

### 2.4. Isolation of Neutrophils

Peripheral blood was collected in EDTA-anticoagulated tubes and slowly laid on the surface of Ficoll (GE Healthcare; Piscataway, NJ, USA), followed by gradient centrifugation at 2000 rpm at 20°C. The lowest layer was collected, and neutrophils were obtained after incubating with a red blood cell lysis buffer (BD Biosciences; San Diego, CA, USA). Cells were cultured with Fc block antibody (BioLegend) in FACS buffer for 10 min to block nonspecific binding, followed by staining with specific cell surface antibodies at 4°C for 30 min. Primary antibodies used in this study included PE-conjugated anti-CD66b (BioLegend) and APC-CY7-conjugated anti-Live/Dead (Life Invitrogen; Carlsbad, CA, USA). Data were acquired on a BD FACSCanto II (BD Biosciences) and further analyzed with FlowJo 10.0 (Tree Star; Ashland, OR, USA) (Supplementary Figure 1). Apoptosis analysis was performed as follows. Neutrophils were collected and stimulated with indicated stimuli. Cells were then stained with PI (BioLegend) and APC-conjugated Annexin V (BioLegend) for 15 min at room temperature. Data were then acquired on BD FACSCanto II (BD Biosciences).

The isolation of neutrophils from the bone marrow of mice was performed using a murine neutrophil isolation kit (130-097-658, Miltenyi; Bergisch Gladbach, Germany) according to the instruction of the manufacturer.

### 2.5. Immunofluorescence Staining

Fresh intestinal biopsies from IBD patients and healthy donors were fixated with 10% paraformaldehyde (PFA) for 24 hours and embedded with optimal cutting temperature compound (OCT) followed by slicing to 5 *μ*m thick sections. OCT-embedded intestinal mucosal tissue sections (5 *μ*m) were dried, followed by incubation of phosphate-buffered saline with Tween-20 (PBS-T). After 3 washes with PBS buffer supplemented with 5% donkey serum, 3% BSA, and 0.1% Triton-X-100 to block nonspecific proteins, the sections were incubated with primary goat anti-MCPIP-1 antibody (1 : 250, Santa Cruz; Dallas, TX, USA) and primary rabbit anti-MPO antibody (1 : 100, Abcam; Cambridge, MA, USA) at 4°C overnight. On the next day, the sections were incubated with donkey anti-goat IgG (1 : 800, Alexa Fluor® 488) and donkey anti-rabbit IgG (1 : 800, Alexa Fluor® 594) at room temperature for 1 hour. After 3 washes, the sections were stained with Hoechst 33342 (1 : 1000, MCE) and mounted with cover slips. Sections were observed with an immunofluorescence microscope (DFC7000T, Leica; Wetzlar, Germany). Additionally, we treated sections with PBS instead of primary antibody as a negative control. The slides were read blindly without any code to avoid observer bias.

### 2.6. Western Blotting Analysis

Neutrophils were lysed by phenylmethylsulfonyl fluoride (PMSF, 1 mM) and radioimmunoprecipitation assay lysis (RIPA) buffer. After centrifugation, the total protein was obtained. Samples were then resolved on sodium dodecyl sulphate-polyacrylamide gel electrophoresis (SDS-PAGE, Epizyme; Shanghai, China) by standard procedure. Western blotting was performed as described previously [32]. Immunoblotting was performed with human primary antibodies to MCPIP-1 (Santa Cruz), inositol-requiring enzyme 1-*α* (IRE1-*α*, 1 : 100, Servicebio; Wuhan, China), protein kinase R-like endoplasmic reticulum kinase (PERK, 1 : 100, Servicebio), binding-immunoglobulin protein (BIP, 1 : 100, Servicebio), p65 (1 : 100, Servicebio), and *β*-actin (Abcam). For signal detection, the Odyssey Infrared Imaging System and Image Studio (LI-COR Biosciences; Lincoln, NE, USA) were used. ImageJ (National Institutes of Health; Bethesda, MD, USA) was used for quantification.

### 2.7. Quantitative Real-Time PCR

Total RNA of neutrophils was extracted with TRIzol (Life Technologies; Carlsbad, CA, USA). The concentration and purity of RNA were determined by a NanoVue spectrophotometer (GE Healthcare), and the quality and quantity of RNA of each sample were assessed through the NanoDrop 2000 (Quawell; Waltham, MA, USA) with an A260/A280 ratio of >1.8 and <2.0 for samples. We synthesized cDNA from 400 ng of RNA using an all-in-one reverse transcription (RT) reagent kit (ABM; Richmond, BC, Canada). PCR was performed using a SYBR Green PCR kit (Takara; Dalian, China) in the ABI prism 7900HT sequence detector (Applied Biosystems; Foster City, CA, USA). RT-PCR reaction conditions were as follows: 95°C for 1 min, 95°C for 15 s, and 60°C for 30 s, repeated for 40 cycles. All primers were synthesized by Sangon BioTech (Shanghai, China), and GAPDH was used as the housekeeping gene. qRT-PCR analysis was calculated with the 2^−*ΔΔ*Ct^ method [19].

### 2.8. ELISA

The procedure of ELISA was preformed according to the manufacturer's instruction (BioLegend). In brief, captured antibodies were incubated in 96-well plates at 4°C overnight. Nonspecific antigens were blocked with assay diluents. The standard and samples were added and incubated at 37°C for 2 hours. After thoroughly washing with 0.05% Tween-PBS, the plates were incubated with detection antibodies for 1 hour and HRP for 30 min. Finally, the color was developed with tetramethylbenzidine (TMB), and the value of OD was detected at 450 nm in Epoch (BioTek; Winooski, VT, USA).

### 2.9. Transwell Assay

Neutrophils (1 × 10^5^) were resuspended in RPMI-1640 medium and added into the upper room of an 8 *μ*m Transwell plate (for human neutrophils) or a 5 *μ*m Transwell plate (for murine neutrophils). The lower room was added with 100 *μ*L of N-Formyl-Met-Leu-Phe (fMLP, 50 nM). Neutrophils were extracted after 3 hours of culture. The medium in the lower room was abandoned after centrifugation (350 g, 10 min). All plates were fixed by 4% PFA, stained by 0.1% crystal violet, and blotted carefully after 2 washes with PBS. The plates were finally observed under the inverted microscopy (DMi1, Leica).

### 2.10. Statistical Analysis

All data were expressed as mean ± SEM and analyzed using Prism V.6.0 software (GraphPad software; San Diego, CA, USA) and SPSS V.20.0 (SPSS; Chicago, IL, USA). Statistical comparisons were performed using an unpaired two-tailed Student's *t*-test for 2 groups and one-way analysis of variance (ANOVA) for more than 2 groups. ^∗^*P* < 0.05, ^∗∗^*P* < 0.01, and ^∗∗∗^*P* < 0.001 were considered to be statistically significant.

## 3. Results

### 3.1. MCPIP-1 Is Highly Increased in Neutrophils of IBD Patients

**Figure 1:**
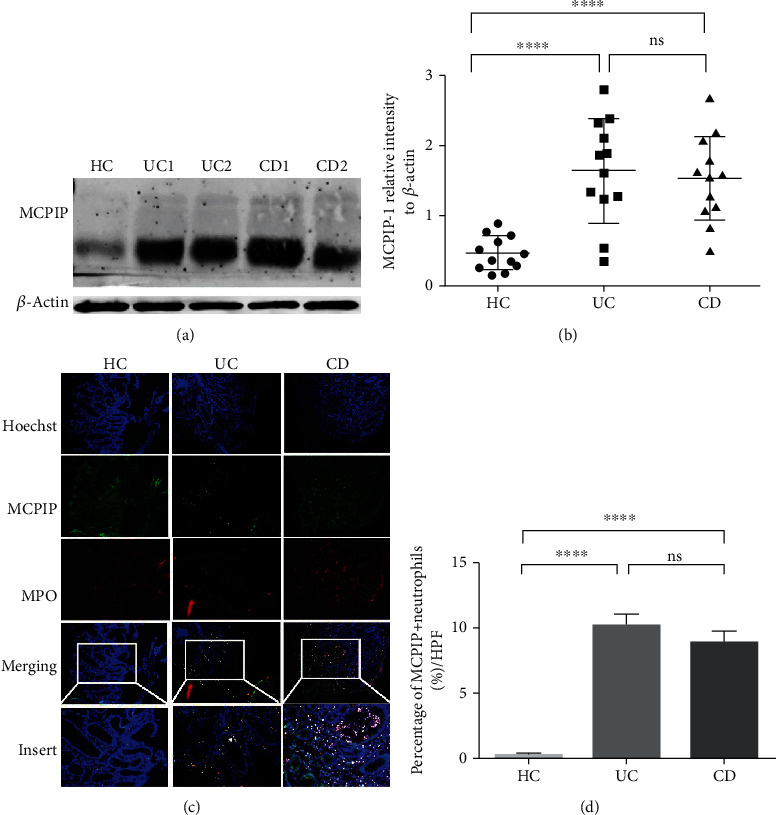
MCPIP-1 is highly increased in neutrophils of IBD patients. Peripheral neutrophils were isolated from patients with active CD (*n* = 12) or active UC (*n* = 12) and healthy donors (HC, *n* = 12). The protein levels of MCPIP were determined by Western blotting (a) and quantified in gray value (b). ^∗∗∗∗^*P* < 0.0001. Abbreviation: ns, not significant. (c) Representative images of double immunofluorescence staining for MCPIP-1 (green) and myeloperoxidase (MPO, red) expression in inflamed colon from an active CD and an active UC patient, and normal colon mucosa of a HC. The arrows indicate double-positive cells after merging (original magnification ×100 and insert ×200). (d) The histogram represents the percentage of double-positive cells in lamina propria of intestinal mucosa from HC (*n* = 10), UC (*n* = 10), and CD (*n* = 10) patients per high-power field (HPF). ^∗∗∗∗^*P* < 0.0001. Abbreviation: ns, not significant.

We first determined the expression of MCPIP-1 in the intestinal mucosa of patients with IBD and heathy donors by qRT-PCR and found that the expression of MCPIP-1 was higher in the intestinal mucosa of patients with IBD compared to heathy donors (Supplementary Figure 2(a)). We then did phenotypic analysis of MCPIP-1 expression in different immune cells. To this end, different immune cells (e.g., B cells, CD4^+^ T cells, monocytes, neutrophils, macrophages, and DCs) were isolated from the peripheral blood and lamina propria of the colon mucosa of healthy controls, and determined the expression of MCPIP-1 by qRT-PCR. We found that MCPIP-1 was mainly expressed in CD4^+^ T cells, monocytes, macrophages, and neutrophils from peripheral blood (*n* = 10) and intestinal mucosa (*n* = 9), especially in neutrophils (Supplementary Figures 2(b) and (c)). We then analyzed the level of MCPIP-1 expression in neutrophils from patients with IBD and healthy donors by Western blotting and observed that the expression of MCPIP-1 was significantly increased in neutrophils of peripheral blood from patients with IBD compared to healthy controls (Figures 1(a) and 1(b)). To localize MCPIP-1 expression in inflamed mucosa, the colon biopsies were collected from patients with active IBD and HC and stained for MCPIP-1 and MPO, a marker of neutrophils, by immunofluorescence staining (Figures 1(c) and 1(d)). We found that MCPIP-1-positive neutrophils were sharply increased in the inflamed colon of patients with active IBD compared to those in HC. Collectively, these data indicate that MCPIP-1 is highly increased in the inflamed mucosa of patients with IBD and mainly expressed in neutrophils.

### 3.2. MCPIP-1 Suppresses the Production of ROS and MPO by Neutrophils from IBD Patients

**Figure 2:**
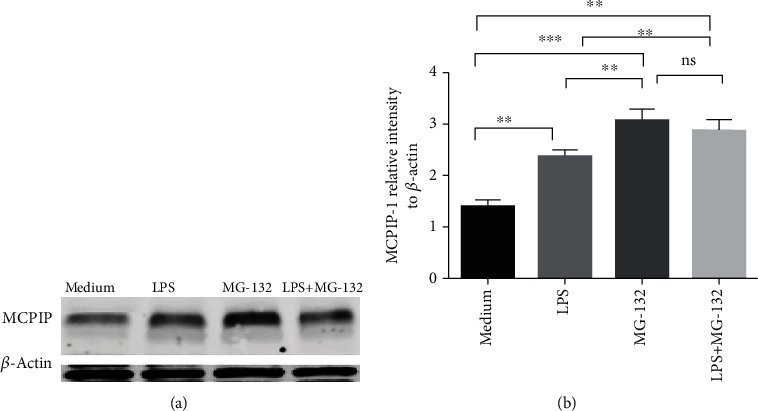
MG-132 facilitates expression of MCPIP-1 in neutrophils. Peripheral neutrophils (1 × 10^6^) were isolated from HC (*n* = 8) and stimulated *in vitro* in the absence (medium alone) or presence of LPS (100 ng/mL), MG-132 (20 *μ*M), and LPS (100 ng/mL) together with MG-132 (20 *μ*M) for 3 hours. Protein was extracted from these cells, and expression of MCPIP was determined by Western blotting (a) and quantified in gray value (b). ^∗^*P* < 0.05 and ^∗∗^*P* < 0.01. Abbreviation: ns, not significant.

To determine the role of MCPIP-1 in regulating neutrophil functions, we cultured neutrophils from patients with active IBD and healthy controls *in vitro* and induced them to overexpress MCPIP-1 by MG-132, a proteasome inhibitor that induces overexpression of MCPIP-1 in several cell types by reducing the degradation of MCPIP-1 [26, 33]. We confirmed that MG-132 markedly promoted neutrophils to express MCPIP-1 (Figures 2(a) and 2(b)). Furthermore, MG-132 has been reported to involve the regulation of neutrophil apoptosis which may initiate the functional changes of neutrophils [34]. Therefore, we performed the apoptosis analysis of neutrophils in the presence of MG-132 at different concentrations for 3 hours. We found that MG-132 did not influence the apoptosis of neutrophils at a low concentration (≤20 *μ*M) (Supplementary Figures 3(a) and (b)). Since MG-132 has also been proven to induce ER stress in many cell types (e.g., rat alveolar macrophages, renal angiomyolipoma cells, and human squamous lingual carcinoma cells) [35–37], we further determined whether MCPIP-1 could modulate ER stress in neutrophils. To this end, we performed the WB analysis of ER stress in the neutrophils with MG-132 for 3 hours and found that MG-132 did not influence the expression of specific markers of ER stress (e.g., inositol-requiring enzyme 1-*α* (IRE1-*α*), protein kinase R-like endoplasmic reticulum kinase (PERK), and binding-immunoglobulin protein (BIP)) in neutrophils (Figures S4 (A)–(D)). However, MG-132 as a proteasome inhibitor was found to markedly inhibit the expression of p65 (Figures S4(A) and (E)), which was in line with other cell types [38, 39]. These results suggest that MG-132 may play an important role as a proteasome inhibitor rather than an inducer of ER stress in neutrophils.

**Figure 3:**
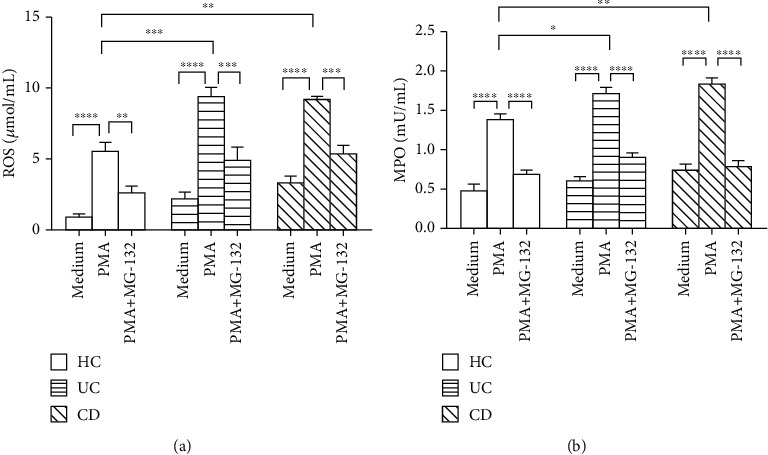
MCPIP-1 downregulates the production of ROS and MPO. Peripheral neutrophils (1 × 10^5^) were isolated from patients with active CD (*n* = 10), active UC (*n* = 10), and HC (*n* = 10) and stimulated *in vitro* with PMA (100 ng/mL) in the absence or presence of MG-132 (20 *μ*M) for 3 hours. The levels of ROS (a) and MPO (b) were measured by an Amplex Red Hydrogen Peroxide Assay Kit according to the manufacturer's instructions. ^∗^*P* < 0.05, ^∗∗^*P* < 0.01, ^∗∗∗^*P* < 0.001, and ^∗∗∗∗^*P* < 0.0001.

Since neutrophils are regarded as important effector cells of the innate immune system to play an essential role in resisting to the invading pathogens, we then investigated whether the functions of neutrophils might be altered when MCPIP-1 was overexpressed. The main way of neutrophils to delete microbes includes the release of ROS and MPO [40]. We measured ROS and MPO production in peripheral neutrophils under spontaneous or PMA-stimulated conditions by the Amplex Red assay and found that the levels of ROS and MPO were significantly increased in neutrophils when stimulated with PMA (Figures 3(a) and 3(b)) and that IBD neutrophils produced more ROS and MPO compared to healthy controls. On the contrary, the production of ROS and MPO by IBD neutrophils was more sharply decreased in the presence of MG-132 compared with controls (Figures 3(a) and 3(b)). Collectively, these data indicate that MCPIP-1 significantly inhibits IBD neutrophils to produce ROS and MPO, which may compose the defense of the intestine to resist against intestinal infection in IBD.

### 3.3. MCPIP-1 Downregulates the Production of Proinflammatory Cytokines in Neutrophils from IBD Patients

**Figure 4:**
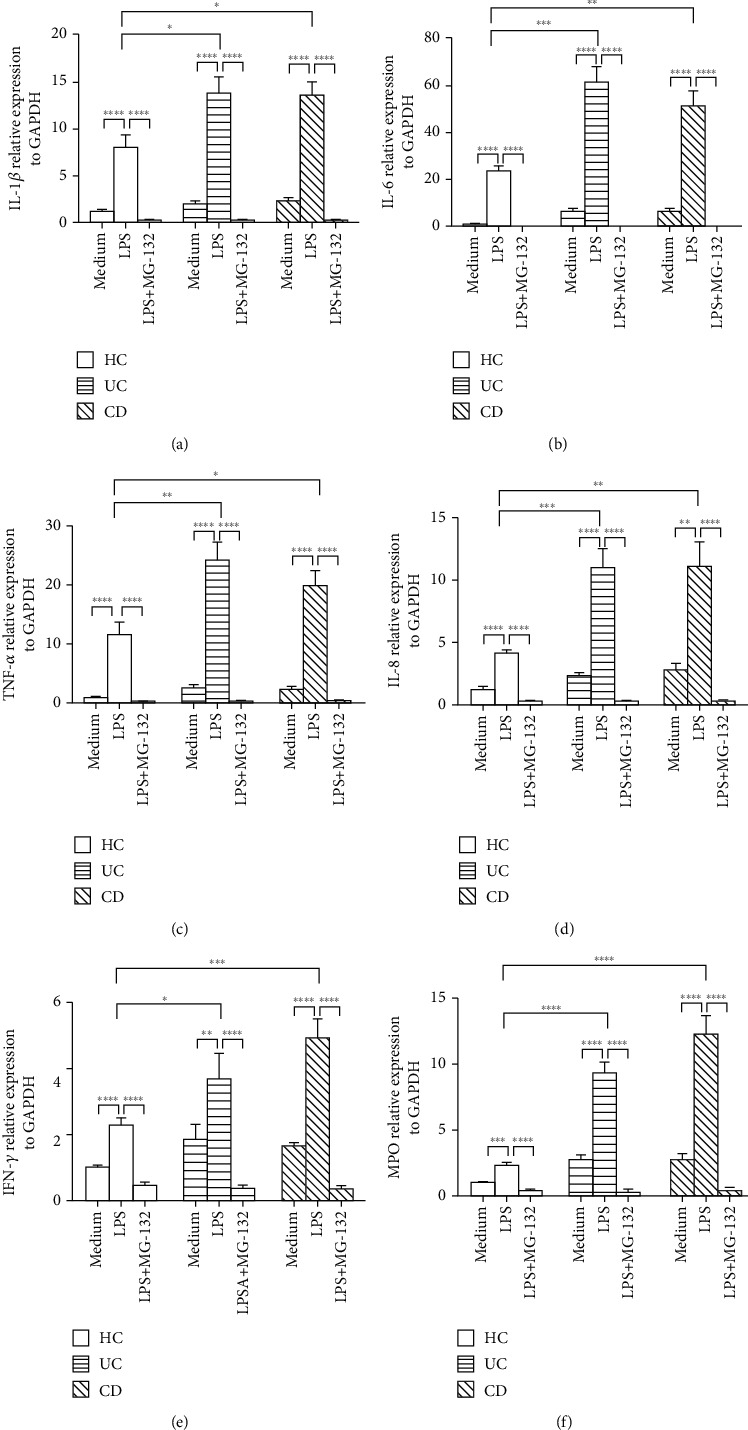
MCPIP-1 degrades the mRNA levels of proinflammatory cytokines in neutrophils. Peripheral neutrophils (5 × 10^6^) were isolated from patients with active CD (*n* = 10), active UC (*n* = 10), and HC (*n* = 10) and stimulated *in vitro* with LPS (100 ng/mL) in the absence or presence of MG-132 (20 *μ*M) for 3 hours. Cells were then harvested, and expression of IL-1*β* (a), IL-6 (b), TNF-*α* (c), IL-8 (d), IFN-*γ* (e), and MPO (f) was analyzed by quantitative RT-PCR and normalized to GAPDH. ^∗^*P* < 0.05, ^∗∗^*P* < 0.01, ^∗∗∗^*P* < 0.001, and ^∗∗∗∗^*P* < 0.0001.

Given that MCPIP-1, which is known to be an endonuclease, degrades the mRNA levels of proinflammatory cytokines (e.g., IL-6, IL-1*β*, and TNF-*α*) in macrophages, we asked whether MCPIP-1 could degrade the mRNA expression of proinflammatory cytokines in IBD neutrophils. As shown in Figure 4, neutrophils were isolated from the peripheral blood of patients with active IBD and healthy donors and stimulated with LPS in the absence or presence of MG-132. As expected, the mRNA levels of IL-6, IL-1*β*, TNF-*α*, IL-8, and IFN-*γ* were found to be significantly higher in IBD neutrophils compared with controls when stimulated with LPS (Figures 4(a)–4(e)). However, the levels of these proinflammatory cytokines were found to be undetectable when MG-132 was present (Figures 4(a)–4(e)). Intriguingly, quantitative RT-PCR further confirmed that the mRNA levels of MPO, an important antibacterial enzyme that is produced by neutrophils, also significantly decreased in IBD neutrophils compared with healthy controls in the presence of MG-132 (Figure 4(f)).

**Figure 5:**
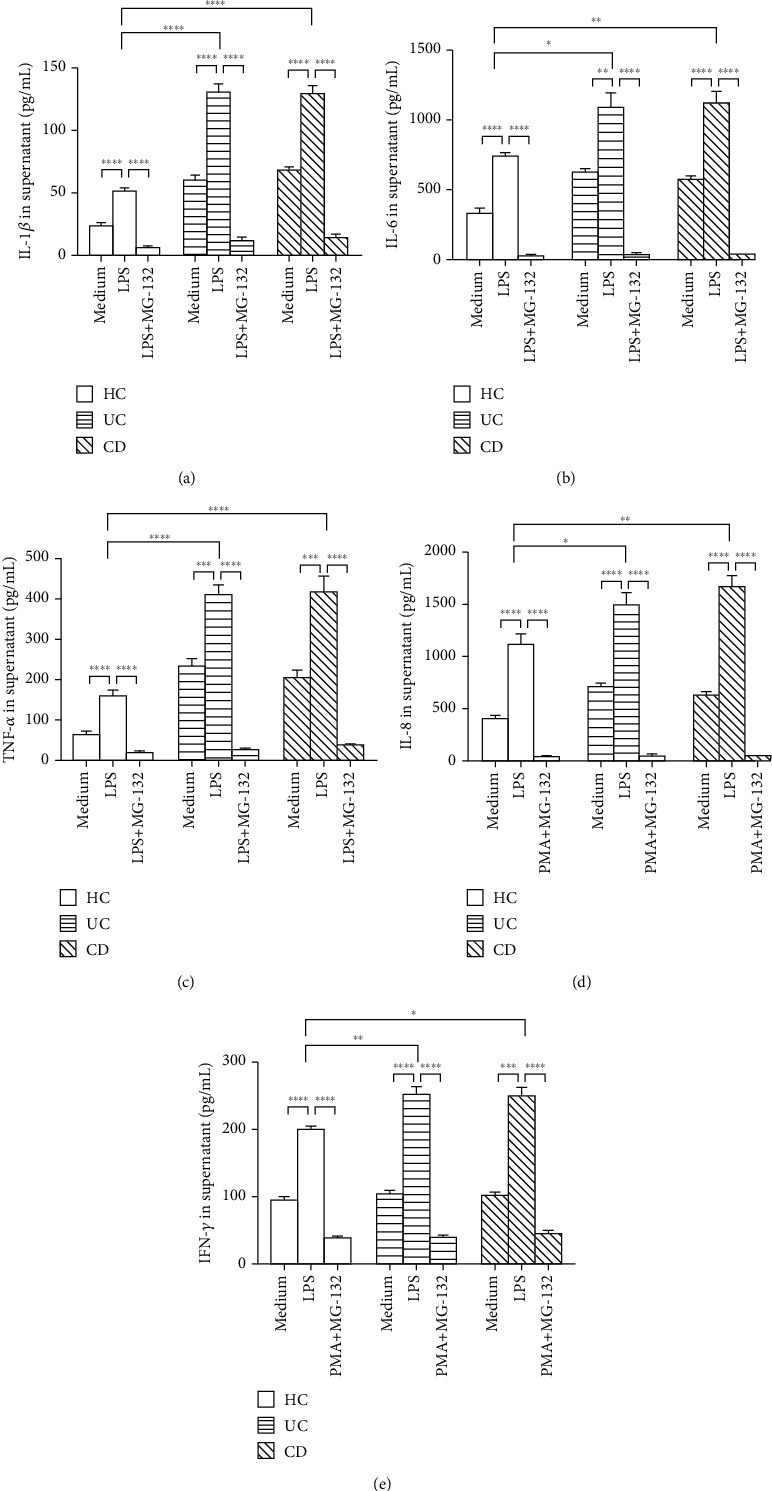
MCPIP-1 degrades the protein levels of proinflammatory cytokine in neutrophils. Peripheral neutrophils (2 × 10^6^) were isolated from patients with active CD (*n* = 10), active UC (*n* = 10), and HC (*n* = 10) and stimulated *in vitro* with LPS (200 ng/mL) in the absence or presence of MG-132 (20 *μ*M) for 3 hours as indicated in Figure 4. Culture medium was replenished, and cells were incubated for another 24 hours. Supernatants were then harvested, and protein levels of IL-1*β* (a), IL-6 (b), TNF-*α* (c), IL-8 (d), and IFN-*γ* (e) were measured by ELISA. ^∗^*P* < 0.05, ^∗∗^*P* < 0.01, ^∗∗∗^*P* < 0.001, and ^∗∗∗∗^*P* < 0.0001.

We then detected the protein levels of IL-6, IL-1*β*, TNF-*α*, IL-8, and IFN-*γ* using ELISA. In line with the results of mRNA levels, the protein levels of IL-6, IL-1*β*, TNF-*α*, IL-8, and IFN-*γ* were significantly increased in the supernatants of IBD neutrophils compared with healthy controls when stimulated with LPS (Figures 5(a)–5(e)). However, they were markedly decreased when stimulated with MG-132 *in vitro* (Figures 5(a)–5(e)). Therefore, these results indicate that MCPIP-1 markedly restricts the production of proinflammatory cytokines in IBD neutrophils.

### 3.4. MCPIP-1 Blocks the Migration of Neutrophils from IBD Patients

**Figure 6:**
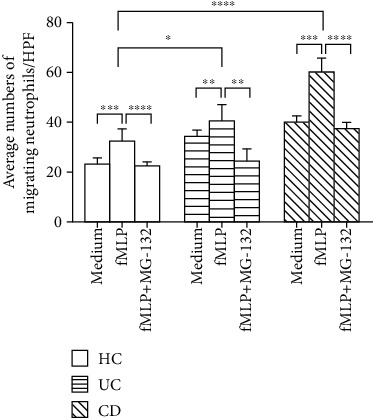
MCPIP-1 suppresses the migration of neutrophils. Peripheral neutrophils (5 × 10^5^) were isolated from HC (*n* = 10), active CD (*n* = 10) and active UC (*n* = 10) patients and measured with an 8 *μ*m Transwell plate under attraction with fMLP (50 nM) in the absence or presence of MG-132 (20 *μ*M) for 3 hours. The histogram represents the number of migrating neutrophils per high-power field (HPF). ^∗∗^*P* < 0.01, ^∗∗∗^*P* < 0.001, and ^∗∗∗∗^*P* < 0.0001.

Under inflammatory conditions, neutrophils migrate into inflamed mucosa via chemotactic signals, such as IL-8, and chemokine (C-X-C motif) ligand-1 (CXCL-1) [7]. Several lines of evidence have confirmed that huge amounts of neutrophils infiltrate into the inflamed mucosa during the early stage of active IBD, particularly in UC [18]. Therefore, we sought to determine the effects of MCPIP-1 on the migration of neutrophils. To this end, peripheral neutrophils were isolated from IBD patients and healthy controls and added into the upper room of an 8 *μ*m Transwell plate to examine the capacity of migration using a Transwell assay. We found that the capacity of migration of IBD neutrophils was enhanced compared with controls when stimulated with fMLP, while it was weakened in the presence of MG-132 (Figure 6). The results indicate that MCPIP-1 potently suppresses the migration of IBD neutrophils.

### 3.5. Overexpression of MCPIP-1 Inhibits the Production of ROS, MPO, and Proinflammatory Cytokines and the Migration of Neutrophils

**Figure 7:**
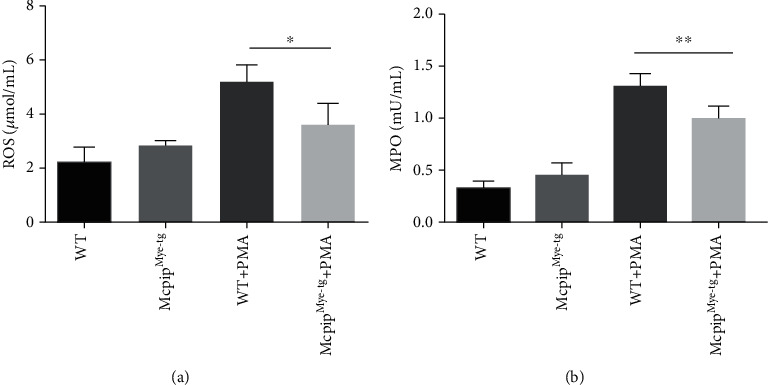
Overexpression of MCPIP-1 inhibits the production of ROS and MPO in neutrophils. Neutrophils (1 × 10^5^) were isolated from the bone marrow of Mcpip^Mye-tg^ (*n* = 6) and WT (*n* = 6) mice. The levels of ROS (a) and MPO (b) were measured by an Amplex Red Hydrogen Peroxide Assay Kit according to the manufacturer's instructions after being stimulated *in vitro* with PMA (100 ng/mL) for 3 hours. ^∗^*P* < 0.05 and ^∗∗^*P* < 0.01.

**Figure 8:**
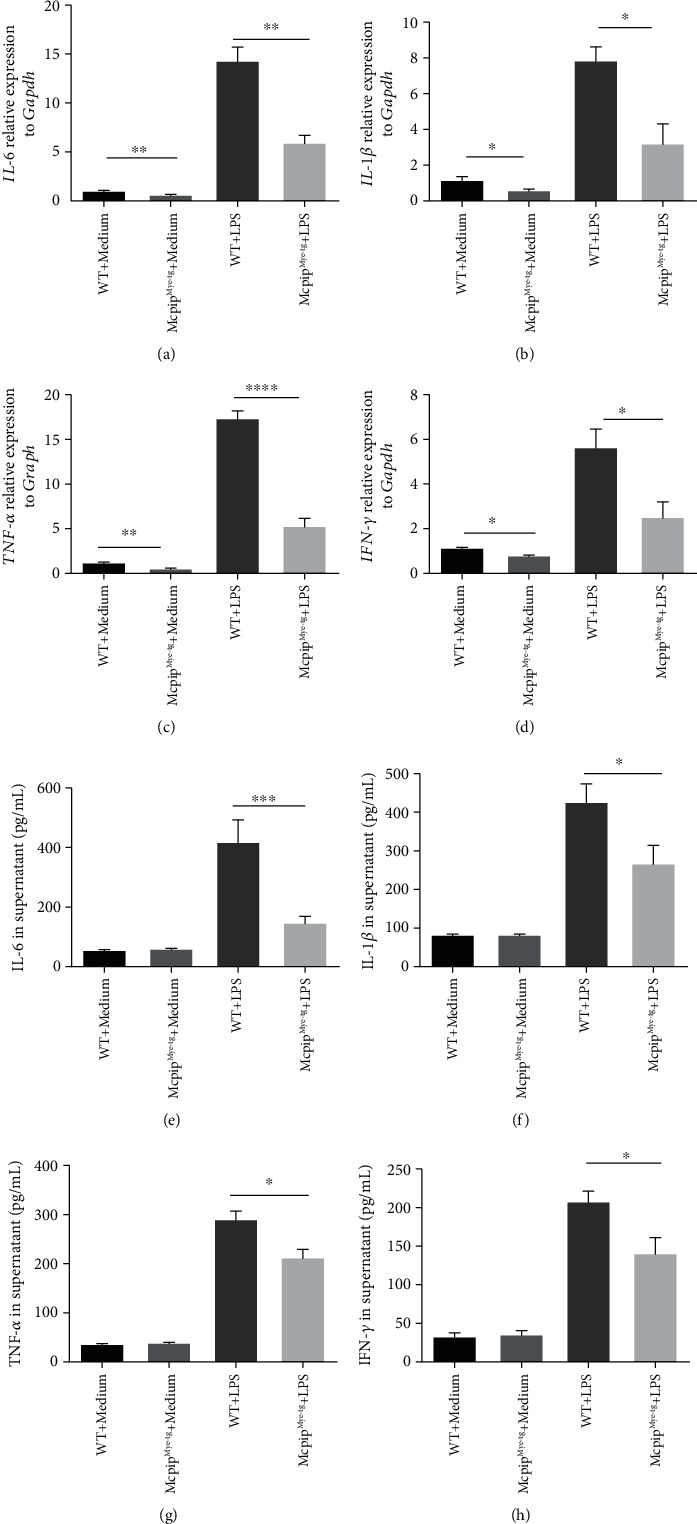
Overexpression of MCPIP-1 degrades the mRNA and protein levels of proinflammatory cytokines in neutrophils. Neutrophils (6 × 10^6^) were isolated from the bone marrow of Mcpip^Mye-tg^ (*n* = 6) and WT (*n* = 6) mice and stimulated with LPS (100 ng/mL) *in vitro* for 3 hours. One part of the neutrophils (5 × 10^6^) was harvested, and expression of *IL-6* (a), *IL-1β* (b), TNF-*α* (c), and IFN-*γ* (d) was analyzed by quantitative RT-PCR and normalized to GAPDH. The rest of the cells (1 × 10^6^) was collected and incubated in the replenished medium for another 24 hours. Supernatants were then harvested, and protein levels of IL-6 (e), IL-1*β* (f), TNF-*α* (g), and IFN-*γ* (h) were measured by ELISA. ^∗^*P* < 0.05, ^∗∗^*P* < 0.01, ^∗∗∗^*P* < 0.001, and ^∗∗∗∗^*P* < 0.0001.

**Figure 9:**
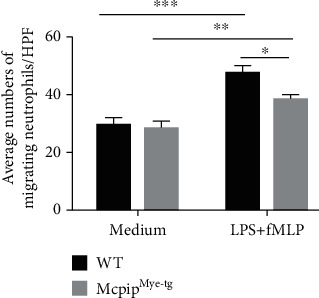
Overexpression of MCPIP-1 blocks the migration of neutrophils. Neutrophils (5 × 10^6^) were isolated from the bone marrow of Mcpip^Mye-tg^ (*n* = 6) and WT (*n* = 6) mice and measured with a 5 *μ*m Transwell plate under attraction with fMLP (50 nM) in the presence of LPS (100 ng/mL) for 3 hours. The histogram represents the number of migrating neutrophils per high-power field (HPF). ^∗∗^*P* < 0.01 and ^∗∗∗^*P* < 0.001.

To further clarify whether MG-132 exerts the dominant effects on neutrophils via MCPIP-1, we isolated neutrophils from the bone marrows of Mcpip^Mye-tg^ and WT mice and stimulated with or without PMA *in vitro* to determine the role of MCPIP-1 in modulating the functions of neutrophils. As shown in Figures 7(a) and 7(b), the production of ROS and MPO was found to be increased in neutrophils from both Mcpip^Mye-tg^ and WT mice when stimulated with PMA *in vitro*. However, the levels of ROS and MPO of Mcpip^Mye-tg^ neutrophils were statistically lower than those of WT controls. We also found that the levels of IL-6, IL-1*β*, TNF-*α*, and IFN-*γ* were decreased in Mcpip^Mye-tg^ neutrophils when stimulated with LPS *in vitro* compared to WT controls (Figures 8(a)–8(h)). Moreover, the migration of Mcpip^Mye-tg^ neutrophils was observed to be compromised compared to WT controls (Figure 9). These results indicate that the same functional changes of Mcpip^Mye-tg^ neutrophils are present as observed in neutrophils treated by MG-132 *in vitro*, suggesting that MG-132 may to some extent exert the effect on neutrophils via MCPIP-1.

## 4. Discussion

As the most abundant innate immune cells in circulation, neutrophils play a vital role in the innate immune system and the maintenance of intestinal homeostasis. Neutrophils are considered to act as double-edged swords as they play both pathological and beneficial roles in intestinal mucosal immunity. A previous study has shown that depletion of neutrophils could promote the experimental colitis in mice [41]. Increasing lines of evidence have illustrated that excessive infiltration of neutrophils and release of inflammatory mediators (e.g., ROS, NETs, and cytokines) involve the progression of intestinal damage, particularly in IBD. Consistently, the infiltration and activation of neutrophils are markedly increased in the peripheral blood and inflamed mucosa from patients with IBD [18, 19], and pathogenic bacteria, bacterial toxin, and proinflammatory cytokines are also present in the inflamed intestinal mucosa and sera of IBD patients, which act as activators to neutrophils [42]. Therefore, an intensive investigation on the potential roles of neutrophils in regulating mucosal immune response will allow us to better understand the pathogenesis of IBD.

To date, MCPIP-1 has been found to be increased in a variety of immune cells (e.g., macrophages and CD4^+^ T cells) under inflammatory conditions. However, the role of MCPIP-1 in regulating IBD neutrophils is still unclear. In the current study, we did find that the levels of MCPIP-1 were increased in neutrophils from both the peripheral blood and inflamed mucosa of IBD patients compared with healthy controls and observed that MCPIP-1 suppressed the production of ROS, MPO, and proinflammatory cytokines and inhibited the migration of IBD neutrophils. Therefore, the present results indicate that MCPIP-1 is increased in neutrophils under inflammatory conditions like IBD and that such an increase of MCPIP-1 expression in IBD neutrophils allows us to further explore the potential roles of MCPIP-1 in the progression of IBD.

In contact with invading pathogens, the classical ways of neutrophils to eliminate invading microorganisms include the engulfment, release of antibacterial peptides (e.g., cathepsins, defensins, lactoferrin, and lysozyme) from granules, and production of ROS. ROS have long been considered to be associated with host defense, while the excessive production of ROS may be related to tissue damage [16]. Our results demonstrated that the capacities of producing ROS in IBD neutrophils decreased when MCPIP-1 was overexpressed. As an inhibitor, MCPIP-1 suppresses the activity of NF-*κ*B. In the current study, we proved that ROS, a product related to the NF-*κ*B pathway, was markedly restricted by MCPIP-1 (Figure 3(a)). The decrease of releasing S100A8/A9 may be related to the function of the endonuclease in MCPIP-1. As an important antibacterial enzyme, the production of MPO was decreased when MG-132 was added as well. Therefore, these data indicate that MCPIP-1 fine-tunes the homeostasis of neutrophils in gut mucosa, including balancing the protective function against pathogen infection and their detrimental roles in intestinal tissue damage.

Neutrophils produce huge amounts of proinflammatory cytokines, e.g., IL-6, IL-1*β*, TNF-*α*, IL-8, and IFN-*γ*, which participate in the pathogenesis of IBD [17]. MCPIP-1 as an endonuclease degrades the mRNA of proinflammatory cytokines, such as IL-6, IL-1*β*, IL-12, IL-2, and TNF-*α* [21, 23, 24]. We found that both the mRNA and protein levels of IL-6, IL-1*β*, TNF-*α*, IL-8, and IFN-*γ* were eliminated in neutrophils when MCPIP-1 was overexpressed. These data were consistent with the results showing that MCPIP-1 may act on other immune cells [43, 44]. Both IL-8 and fMLP are indispensable chemotactic agents that are crucial for neutrophils to migrate into affected mucosa [45]. As one of the G protein-coupled receptor (GPCR) agonists, fMLP activates NF-*κ*B, MAPK, and PI3K/Akt signaling pathways, which play a crucial role in the production of IL-8 in human neutrophils [46]. Owing to a decrease of IL-8, the migration of neutrophils was then reduced under the conditions of MCPIP-1 overexpression. Thus, these data suggest that MCIPIP-1 downregulates the proinflammatory functions and migration of neutrophils, which may play an important protective role in the pathogenesis of IBD.

In addition, we found that MCPIP-1 could suppress the production of ROS, MPO, and proinflammatory cytokines as well as the migration in IBD patients compared to controls. To investigate the role of MCPIP-1 in regulating neutrophil functions, we induced overexpression of MCPIP-1 in IBD neutrophils by MG-132 and found that MG-132 could induce a high level of MCPIP-1. Interestingly, when stimulated by LPS and MG-132, neutrophils did not produce an excessive level of MCPIP-1 compared to LPS and MG-132. It may be ascribed to accumulation of MCPIP-1 in limiting the synthesis of itself [23]. As a proteasome inhibitor, MG-132 is observed to enhance MCPIP-1 expression in several types of immune cells by inhibiting the degradation of MCPIP-1 [26, 33], and it also inhibits the activity of NF-*κ*B *in vivo* [47]. In addition, a previous study has also reported that MG-132 could alleviate the experimental colitis in mice via mediating the immunoinhibitory effects on CD4^+^ T cells [48]. Otherwise, MG-132 also activates c-Jun N-terminal kinase (JNK1), which initiates the apoptosis [49]. We isolated neutrophils from Mcpip^Mye-tg^ and WT mice to further test the role of MCPIP-1 in modulating neutrophil functions and found that the same functional alterations were present in Mcpip^Mye-tg^ neutrophils as observed in neutrophils treated by MG-132 *in vitro*, suggesting that MG-132 may to some extent exert the effect on neutrophils via MCPIP-1. Therefore, these results indicate that MCPIP-1 could alleviate the activities of neutrophils in IBD and that MG-132 as an inducer of MCPIP-1 overexpression may serve as a potential therapeutic approach in the management of IBD.

## 5. Conclusion

Collectively, we have demonstrated that MCPIP-1 restricts the functions of neutrophils in IBD and that MCPIP-1 downregulates the productions of MPO, ROS, and proinflammatory cytokines and suppresses the migration in IBD neutrophils. Through these studies, we can envisage that targeting MCPIP-1 in neutrophils may be beneficial for treatment of IBD. As a critical trigger of MCPIP-1 for negatively regulating neutrophil activities, MG-132 may be a novel therapeutic approach in the management of human IBD.

## Figures and Tables

**Table 1:** Demographics and clinical characteristics of the study population.

	Blood samples			Intestinal biopsies			
	HC	CD	UC	HC		CD	UC
No. patients	55	68	62	10		14	12
Age (y)	42.3 ± 18.1	25.8 ± 14.1	36.4 ± 15.6	27.5 ± 14.3		26.7 ± 16.8	38.7 ± 17.5

Sex (F/M)	29/26	36/32	36/26	4/6		8/6	5/7
Duration (mo)		42.9 ± 16.3	46.1 ± 39.3			27.6 ± 26.2	22.3 ± 21.9

Current therapy							
5-ASA		19	50			2	9
Immunosuppressants		50	22			11	3
Biologics		17	4			3	0

Disease extent^a^							
E1			7				2
E2			31	3
E3			24	7

Disease location^a^							
L1		11				0	
L2		25	4				
L3		32	10				

Mayo score			5.55 ± 0.54				4.0 ± 0.76

CDAI		168.4 ± 13.5				122.0 ± 18.3	

^a^Montreal classification.

## Data Availability

All data generated or analyzed during this study are included in this article.

## Supplementary Materials

Supplementary 1 Supplementary Figure 1: the purity of isolated peripheral neutrophils. Neutrophils were isolated from the peripheral blood of a patient with active CD, a patient with active UC, and a healthy donor (HC) with Ficoll gradient centrifugation. After depletion of red blood cells, neutrophils were measured with flow cytometry. The sample of neutrophils was selected for further experiments when the purity was greater than 93%.

Supplementary 2 Supplementary Figure 2: MCPIP-1 is expressed in different immune cells in the peripheral blood and intestinal mucosa. (a) Intestinal mucosa biopsies were obtained from active CD (*n* = 14) or active UC (*n* = 12) patients and HC (*n* = 10), and MCPIP-1 was analyzed by quantitative RT-PCR and normalized to GAPDH. (b) Peripheral B cells, CD4^+^ T cells, CD8^+^ T cells, monocytes, and neutrophils were obtained from HC (*n* = 10), and MCPIP-1 was analyzed by quantitative RT-PCR and normalized to GAPDH. ^∗∗^*P* < 0.01 vs. the data from B cells. (c) Intestinal lamina propria CD4^+^ T cells, B cells, dendritic cells (DCs), macrophages, and neutrophils were isolated from normal intestinal mucosa from patients (*n* = 9) who underwent colectomy for colon cancer, and MCPIP-1 was analyzed by quantitative RT-PCR and normalized to GAPDH. ^∗∗^*P* < 0.01 and ^∗∗∗^*P* < 0.001 vs. the data from B cells.

Supplementary 3 Supplementary Figure 3: MG-132 does not affect the apoptosis of neutrophils at low concentrations. (a, b) Peripheral neutrophils were isolated from healthy donors (*n* = 8) and incubated with medium alone, TNF-*α* (20 ng/mL), or MG-132 at different concentrations (10, 20, and 40 *μ*M) in the absence (medium alone) or presence of LPS (100 ng/mL) for 3 hours. Cells were collected and detected for the apoptosis by flow cytometry. ^∗^*P* < 0.05, ^∗∗^*P* < 0.01, and ^∗∗∗^*P* < 0.001 vs. the data from the medium group. Abbreviation: ns, not significant.

Supplementary 4 Supplementary Figure 4: MG-132 does not induce ER stress in neutrophils at low concentrations. (a) Peripheral neutrophils were isolated from healthy donors (*n* = 8) and incubated in medium alone or stimulated with MG-132 (20 *μ*M) in the absence or presence of LPS (100 ng/mL) for 3 hours. Protein was extracted from these cells, and expression of IRE1-*α,* PERK, BIP, and p65 was determined by Western blotting (a) and quantified in gray value (b). ^∗^*P* < 0.05, ^∗∗^*P* < 0.01, and ^∗∗∗^*P* < 0.001 vs. the data from neutrophils cultured in medium alone. Abbreviation: ns, not significant.

## References

[1] Cader M. Z., Kaser A. (2013). Recent advances in inflammatory bowel disease: mucosal immune cells in intestinal inflammation. *Gut*.

[2] Ng S. C., Tang W., Ching J. Y. (2013). Incidence and phenotype of inflammatory bowel disease based on results from the Asia-pacific Crohn's and colitis epidemiology study. *Gastroenterology*.

[3] Sun M., He C., Cong Y., Liu Z. (2015). Regulatory immune cells in regulation of intestinal inflammatory response to microbiota. *Mucosal Immunology*.

[4] Liu Z. (2013). Microbiota regulation of inflammatory bowel disease and colorectal cancer. *Seminars in Cancer Biology*.

[5] Ma C., Wu W., Lin R. (2019). Critical role of CD6highCD4+ T cells in driving Th1/Th17 cell immune responses and mucosal inflammation in IBD. *Journal of Crohn's & Colitis*.

[6] Bilotta A., Cong Y. (2019). Gut microbiota metabolite regulation of host defenses at mucosal surfaces: implication in precision medicine. *Precision Clinical Medicine*.

[7] Kolaczkowska E., Kubes P. (2013). Neutrophil recruitment and function in health and inflammation. *Nature Reviews. Immunology*.

[8] Summers C., Rankin S. M., Condliffe A. M., Singh N., Peters A. M., Chilvers E. R. (2013). Neutrophil kinetics in health and disease. *Trends in Immunology*.

[9] Lehman H. K., Segal B. H. (2020). The role of neutrophils in host defense and disease. *The Journal of Allergy and Clinical Immunology*.

[10] Dupré-Crochet S., Erard M., Nüβe O. (2013). ROS production in phagocytes: why, when, and where?. *Journal of Leukocyte Biology*.

[11] Zygiel E. M., Nolan E. M. (2018). Transition metal sequestration by the host-defense protein calprotectin. *Annual Review of Biochemistry*.

[12] Papayannopoulos V. (2018). Neutrophil extracellular traps in immunity and disease. *Nature Reviews. Immunology*.

[13] Butin-Israeli V., Bui T. M., Wiesolek H. L. (2019). Neutrophil-induced genomic instability impedes resolution of inflammation and wound healing. *The Journal of Clinical Investigation*.

[14] Butin‐Israeli V., Houser M. C., Feng M. (2016). Deposition of microparticles by neutrophils onto inflamed epithelium: a new mechanism to disrupt epithelial intercellular adhesions and promote transepithelial migration. *The FASEB journal*.

[15] Wéra O., Lancellotti P., Oury C. (2016). The dual role of neutrophils in inflammatory bowel diseases. *Journal of clinical medicine*.

[16] Makhezer N., Khemis M. B., Liu D. (2016). NOX1-derived ROS drive the expression of lipocalin-2 in colonic epithelial cells in inflammatory conditions. *Mucosal Immunology*.

[17] Tamassia N., Bianchetto‐Aguilera F., Arruda‐Silva F. (2018). Cytokine production by human neutrophils: revisiting the 'dark side of the moon. *European journal of clinical investigation*.

[18] Zhou G., Yu L., Fang L. (2018). CD177(+) neutrophils as functionally activated neutrophils negatively regulate IBD. *Gut*.

[19] Zhang C., Shu W., Zhou G. (2018). Anti-TNF-therapy suppresses proinflammatory activities of mucosal neutrophils in inflammatory bowel disease. *Mediators of Inflammation*.

[20] Liang J., Wang J., Azfer A. (2008). A novel CCCH-zinc finger protein family regulates proinflammatory activation of macrophages. *The Journal of Biological Chemistry*.

[21] Matsushita K., Takeuchi O., Standley D. M. (2009). Zc3h12a is an RNase essential for controlling immune responses by regulating mRNA decay. *Nature*.

[22] Liang J., Saad Y., Lei T. (2010). MCP-induced protein 1 deubiquitinates TRAF proteins and negatively regulates JNK and NF-kappaB signaling. *The Journal of Experimental Medicine*.

[23] Mizgalska D., Węgrzyn P., Murzyn K. (2010). Interleukin-1-inducible MCPIP protein has structural and functional properties of RNase and participates in degradation of IL-1beta mRNA. *The FEBS Journal*.

[24] Li M., Cao W., Liu H. (2012). MCPIP1 down-regulates IL-2 expression through an ARE-independent pathway. *PLoS ONE*.

[25] Suzuki H. I., Arase M., Matsuyama H. (2011). MCPIP1 ribonuclease antagonizes dicer and terminates microRNA biogenesis through precursor microRNA degradation. *Molecular Cell*.

[26] Uehata T., Iwasaki H., Vandenbon A. (2013). Malt1-induced cleavage of regnase-1 in CD4(+) helper T cells regulates immune activation. *Cell*.

[27] Marona P., Górka J., Mazurek Z. (2017). MCPIP1 downregulation in clear cell renal cell carcinoma promotes vascularization and metastatic progression. *Cancer Research*.

[28] Huang S., Miao R., Zhou Z. (2013). MCPIP1 negatively regulates toll-like receptor 4 signaling and protects mice from LPS-induced septic shock. *Cellular Signalling*.

[29] Iwasaki A., Medzhitov R. (2010). Regulation of adaptive immunity by the innate immune system. *Science*.

[30] He C., Shi Y., Wu R. (2016). miR-301a promotes intestinal mucosal inflammation through induction of IL-17A and TNF-*α* in IBD. *Gut*.

[31] Kapoor N., Niu J., Saad Y. (2015). Transcription factors STAT6 and KLF4 implement macrophage polarization via the dual catalytic powers of MCPIP. *Journal of Immunology*.

[32] Sun M., He C., Wu W. (2017). Hypoxia inducible factor-1*α*-induced interleukin-33 expression in intestinal epithelia contributes to mucosal homeostasis in inflammatory bowel disease. *Clinical and Experimental Immunology*.

[33] Zhang Y., Huang T., Jiang L. (2019). MCP-induced protein 1 attenuates sepsis-induced acute lung injury by modulating macrophage polarization via the JNK/c-Myc pathway. *International immunopharmacology*.

[34] Spirli C., Villani A., Mariotti V., Fabris L., Fiorotto R., Strazzabosco M. (2015). Posttranslational regulation of polycystin-2 protein expression as a novel mechanism of cholangiocyte reaction and repair from biliary damage. *Hepatology*.

[35] Fan T., Huang Z., Chen L. (2016). Associations between autophagy, the ubiquitin-proteasome system and endoplasmic reticulum stress in hypoxia-deoxygenation or ischemia-reperfusion. *European Journal of Pharmacology*.

[36] Siroky B. J., Yin H., Babcock J. T. (2012). Human TSC-associated renal angiomyolipoma cells are hypersensitive to ER stress. *American Journal of Physiology-Renal Physiology*.

[37] Chen H., Ren X., Wang W. (2014). Upregulated ROS production induced by the proteasome inhibitor MG-132 on XBP1 gene expression and cell apoptosis in Tca-8113 cells. *Biomedicine & Pharmacotherapy*.

[38] Zuo Q. P., Liu S. K., Li Z. J. (2011). NF-kappaB p65 modulates the telomerase reverse transcriptase in the HepG_2_ hepatoma cell line. *European journal of pharmacology*.

[39] Skalniak L., Dziendziel M., Jura J. (2014). MCPIP1 contributes to the toxicity of proteasome inhibitor MG-132 in HeLa cells by the inhibition of NF-*κ*B. *Molecular and Cellular Biochemistry*.

[40] Mantovani A., Cassatella M. A., Costantini C., Jaillon S. (2011). Neutrophils in the activation and regulation of innate and adaptive immunity. *Nature Reviews. Immunology*.

[41] Kühl A. A., Kakirman H., Janotta M. (2007). Aggravation of different types of experimental colitis by depletion or adhesion blockade of neutrophils. *Gastroenterology*.

[42] Biskup E., Kamstrup M., Manfé V., Gniadecki R. (2013). Proteasome inhibition as a novel mechanism of the proapoptotic activity of *γ*-secretase inhibitor I in cutaneous T-cell lymphoma. *The British Journal of Dermatology*.

[43] Jura J., Skalniak L., Koj A. (2012). Monocyte chemotactic protein-1-induced protein-1 (MCPIP1) is a novel multifunctional modulator of inflammatory reactions. *Biochimica et Biophysica Acta*.

[44] Cifuentes R. A., Cruz-Tapias P., Rojas-Villarraga A., Anaya J. M. (2010). ZC3H12A (MCPIP1): molecular characteristics and clinical implications. *Clinica Chimica Acta*.

[45] Russo R. C., Garcia C. C., Teixeira M. M., Amaral F. A. (2014). The CXCL8/IL-8 chemokine family and its receptors in inflammatory diseases. *Expert Review of Clinical Immunology*.

[46] Hidalgo M. A., Carretta M. D., Teuber S. E. (2015). fMLP-induced IL-8 release is dependent on NADPH oxidase in human neutrophils. *Journal of immunology research*.

[47] Ma Y., Chen B., Liu D. (2011). MG132 treatment attenuates cardiac remodeling and dysfunction following aortic banding in rats via the NF-*κ*B/TGF*β*1 pathway. *Biochemical Pharmacology*.

[48] Inoue S., Nakase H., Matsuura M. (2009). The effect of proteasome inhibitor MG132 on experimental inflammatory bowel disease. *Clinical and Experimental Immunology*.

[49] Dai Y., Rahmani M., Grant S. (2003). Proteasome inhibitors potentiate leukemic cell apoptosis induced by the cyclin-dependent kinase inhibitor flavopiridol through a SAPK/JNK- and NF-*κ*B-dependent process. *Oncogene*.

