# Supplementary figures and images for: Monocyte Chemotactic Protein 1-Induced Protein 1 Is Highly Expressed in Inflammatory Bowel Disease and Negatively Regulates Neutrophil Activities
Source: Mediators Inflamm. 2020 Dec 22;2020:8812020. doi: 10.1155/2020/8812020 (PMC7803109; doi:10.1155/2020/8812020)

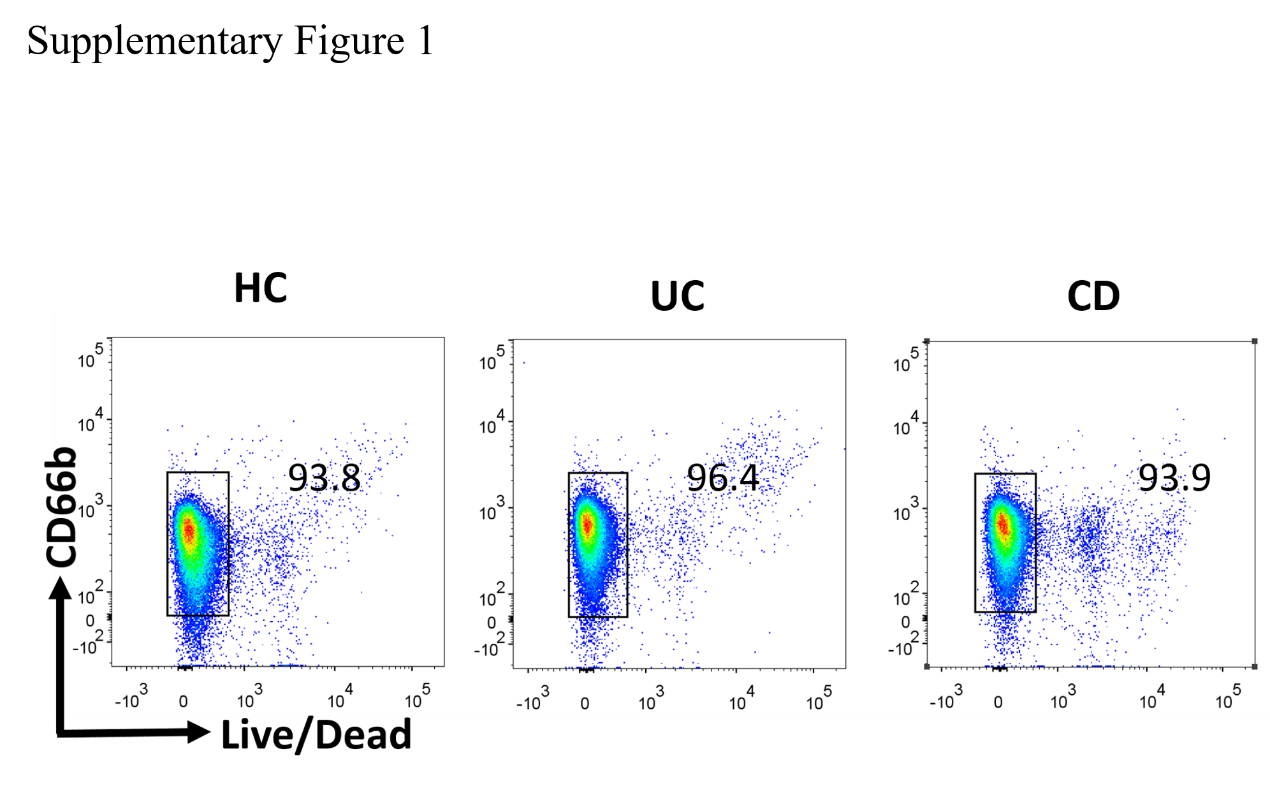

Supplement: Supplementary 1 — Supplementary Figure 1: the purity of isolated peripheral neutrophils. Neutrophils were isolated from the peripheral blood of a patient with active CD, a patient with active UC, and a healthy donor (HC) with Ficoll gradient centrifugation. After depletion of red blood cells, neutrophils were measured with flow cytometry. The sample of neutrophils was selected for further experiments when the purity was greater than 93%. [file 8812020.f1.docx]

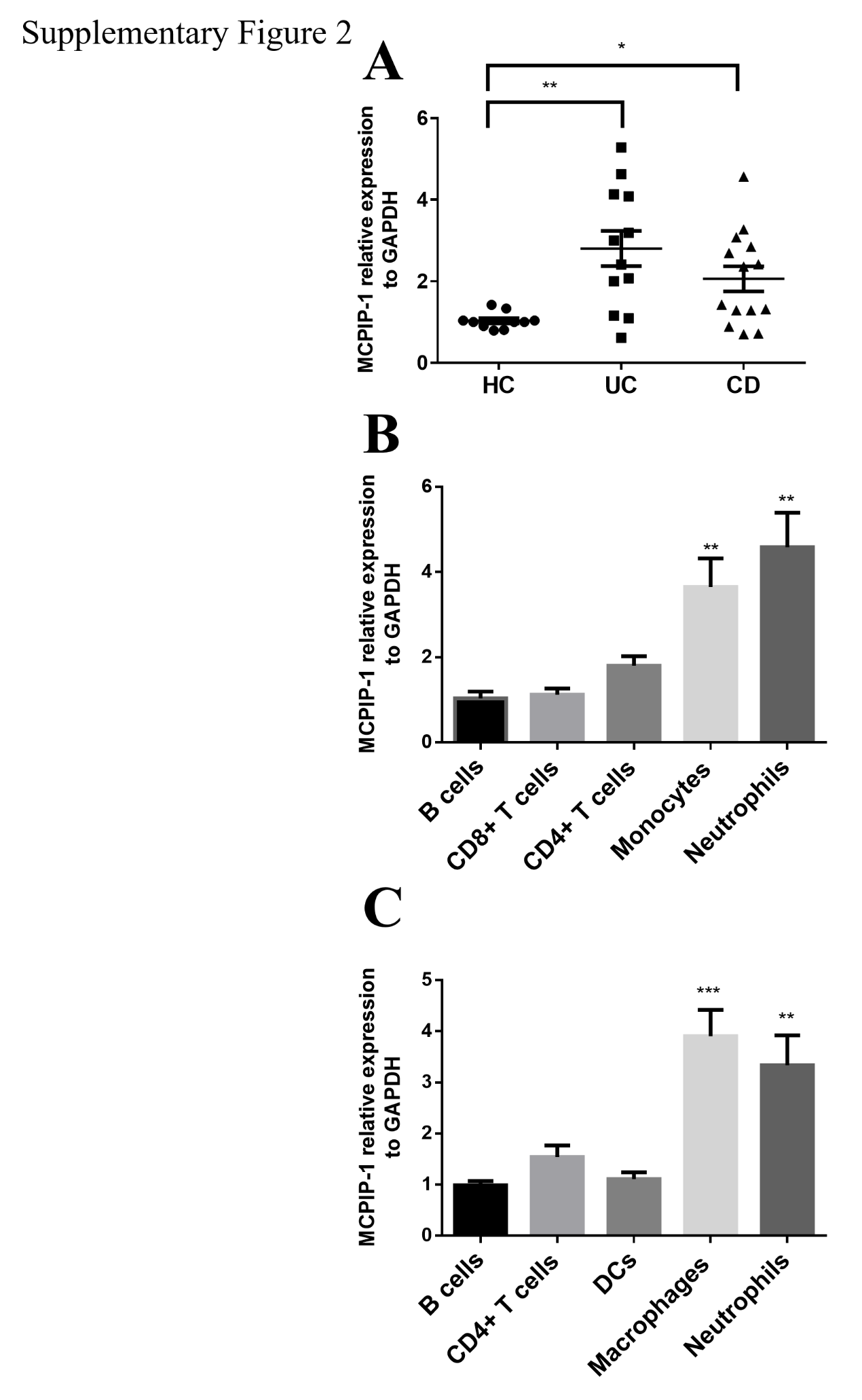

Supplement: Supplementary 2 — Supplementary Figure 2: MCPIP-1 is expressed in different immune cells in the peripheral blood and intestinal mucosa. (a) Intestinal mucosa biopsies were obtained from active CD (n = 14) or active UC (n = 12) patients and HC (n = 10), and MCPIP-1 was analyzed by quantitative RT-PCR and normalized to GAPDH. (b) Peripheral B cells, CD4+ T cells, CD8+ T cells, monocytes, and neutrophils were obtained from HC (n = 10), and MCPIP-1 was analyzed by quantitative RT-PCR and normalized to GAPDH. ∗∗P < 0.01 vs. the data from B cells. (c) Intestinal lamina propria CD4+ T cells, B cells, dendritic cells (DCs), macrophages, and neutrophils were isolated from normal intestinal mucosa from patients (n = 9) who underwent colectomy for colon cancer, and MCPIP-1 was analyzed by quantitative RT-PCR and normalized to GAPDH. ∗∗P < 0.01 and ∗∗∗P < 0.001 vs. the data from B cells. [file 8812020.f2.docx]

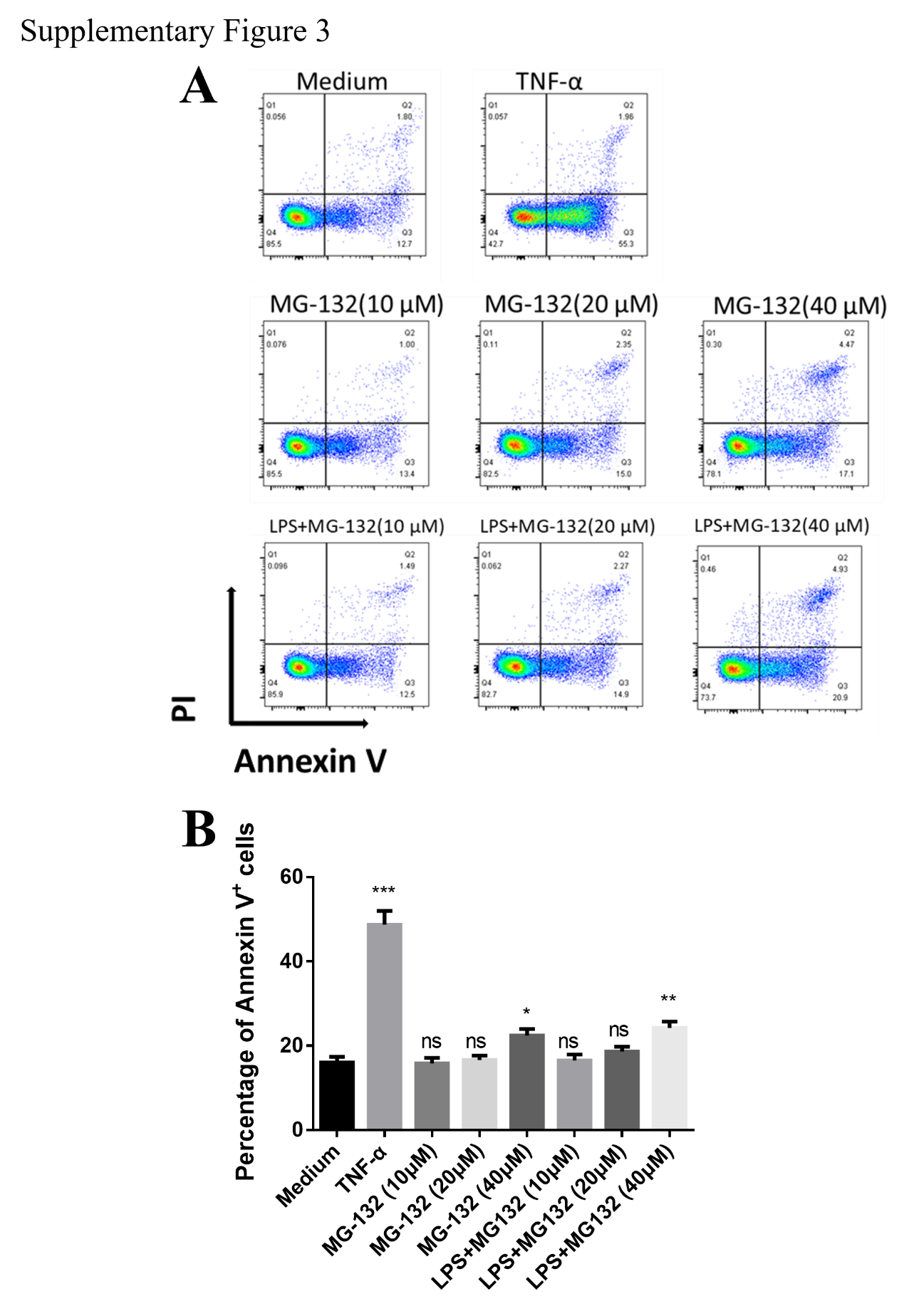

Supplement: Supplementary 3 — Supplementary Figure 3: MG-132 does not affect the apoptosis of neutrophils at low concentrations. (a, b) Peripheral neutrophils were isolated from healthy donors (n = 8) and incubated with medium alone, TNF-α (20 ng/mL), or MG-132 at different concentrations (10, 20, and 40 μM) in the absence (medium alone) or presence of LPS (100 ng/mL) for 3 hours. Cells were collected and detected for the apoptosis by flow cytometry. ∗P < 0.05, ∗∗P < 0.01, and ∗∗∗P < 0.001 vs. the data from the medium group. Abbreviation: ns, not significant. [file 8812020.f3.docx]

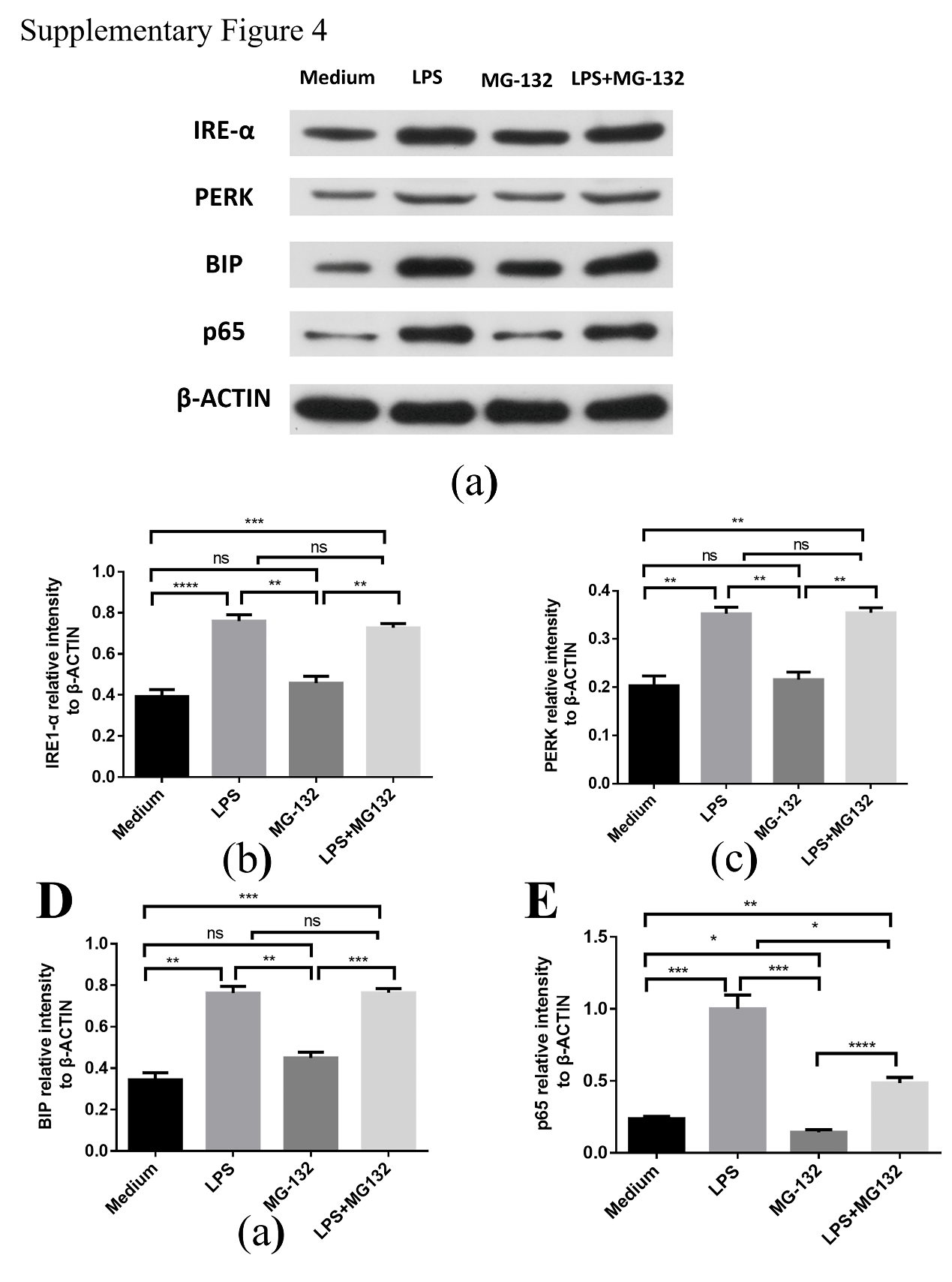

Supplement: Supplementary 4 — Supplementary Figure 4: MG-132 does not induce ER stress in neutrophils at low concentrations. (a) Peripheral neutrophils were isolated from healthy donors (n = 8) and incubated in medium alone or stimulated with MG-132 (20 μM) in the absence or presence of LPS (100 ng/mL) for 3 hours. Protein was extracted from these cells, and expression of IRE1-α, PERK, BIP, and p65 was determined by Western blotting (a) and quantified in gray value (b). ∗P < 0.05, ∗∗P < 0.01, and ∗∗∗P < 0.001 vs. the data from neutrophils cultured in medium alone. Abbreviation: ns, not significant. [file 8812020.f4.docx]
